# Identification of a Novel Human LAP1 Isoform That Is Regulated by Protein Phosphorylation

**DOI:** 10.1371/journal.pone.0113732

**Published:** 2014-12-02

**Authors:** Mariana Santos, Sara C. Domingues, Patrícia Costa, Thorsten Muller, Sara Galozzi, Katrin Marcus, Edgar F. da Cruz e Silva, Odete A. da Cruz e Silva, Sandra Rebelo

**Affiliations:** 1 Laboratório de Neurociências e Sinalização, Centro de Biologia Celular, SACS, Universidade de Aveiro, Aveiro, Portugal; 2 Department of Functional Proteomics, Medical Proteome Center, Ruhr University Bochum, Bochum, Germany; International Centre for Genetic Engineering and Biotechnology, Italy

## Abstract

Lamina associated polypeptide 1 (LAP1) is an integral protein of the inner nuclear membrane that is ubiquitously expressed. LAP1 binds to lamins and chromatin, probably contributing to the maintenance of the nuclear envelope architecture. Moreover, LAP1 also interacts with torsinA and emerin, proteins involved in DYT1 dystonia and X-linked Emery-Dreifuss muscular dystrophy disorder, respectively. Given its relevance to human pathological conditions, it is important to better understand the functional diversity of LAP1 proteins. In rat, the LAP1 gene (*TOR1AIP1*) undergoes alternative splicing to originate three LAP1 isoforms (LAP1A, B and C). However, it remains unclear if the same occurs with the human *TOR1AIP1* gene, since only the LAP1B isoform had thus far been identified in human cells. *In silico* analysis suggested that, across different species, potential new LAP1 isoforms could be generated by alternative splicing. Using shRNA to induce LAP1 knockdown and HPLC-mass spectrometry analysis the presence of two isoforms in human cells was described and validated: LAP1B and LAP1C; the latter is putatively N-terminal truncated. LAP1B and LAP1C expression profiles appear to be dependent on the specific tissues analyzed and in cultured cells LAP1C was the major isoform detected. Moreover, LAP1B and LAP1C expression increased during neuronal maturation, suggesting that LAP1 is relevant in this process. Both isoforms were found to be post-translationally modified by phosphorylation and methionine oxidation and two LAP1B/LAP1C residues were shown to be dephosphorylated by PP1. This study permitted the identification of the novel human LAP1C isoform and partially unraveled the molecular basis of LAP1 regulation.

## Introduction

The eukaryotic nucleus is a complex organelle enclosed by a double membrane, the nuclear envelope (NE). The NE separates the cytoplasm from de the nucleus in eukaryotic cells and is structurally composed by the inner nuclear membrane (INM), the outer nuclear membrane (ONM), the nuclear lamina and the nuclear pore complexes. The perinuclear space is located between the INM and the ONM, however these membranes are joined in some regions at the nuclear pore complexes [1]. The INM contains specific integral membrane proteins [2], [3] and most of them interact with lamins (the main components of the nuclear lamina) and/or chromatin.

One of the first lamin associated proteins identified was the lamina associated polypeptide 1 (LAP1) [4]. LAP1 was initially identified using a monoclonal antibody generated against lamina-enriched fractions of rat liver nuclei. This antibody recognized three rat proteins corresponding to LAP1A, B and C with molecular weights of 75, 68 and 55 kDa, respectively [4]. These proteins are type 2 transmembrane (TM) proteins, comprising a nucleoplasmic N-terminal domain, a single TM domain and a lumenal C-terminal domain, located in the perinuclear space [5]. Moreover, rat LAP1 family members are generated by alternative splicing and differ only in their nucleoplasmic domain. The full-length cDNA of rat LAP1C was isolated from a cDNA expression library prepared from rat liver polyA^+^ mRNA. Additionally, partial clones of LAP1B and LAP1C were isolated. These clones were identical to some sequences of LAP1C cDNA but have two additional insertions [5]. To date, only one isoform had been identified and characterized in human cells and it corresponded to LAP1B. Kondo *et al*, isolated a clone from HeLa cells that was similar to the rat LAP1C cDNA, and encoded a protein with a molecular weight (66.3 kDa) very close to the expected size for rat LAP1B. Therefore, it was concluded that this clone should correspond to the human LAP1B isoform [6]. Additionally, another human variant of LAP1B was identified, but it has only one amino acid (alanine) less [7], [8] than the previously reported LAP1B. Of note, and up to the date of this publication, it remained unclear whether LAP1 is alternatively spliced in human cells, potentially giving rise to other human LAP1 isoforms.

Moreover, the function of LAP1 remains poorly understood. However, it was described that LAP1 binds directly to lamins and indirectly to chromosomes [9]. It is reasonable to deduce that, LAP1 may be involved in the positioning of lamins and chromatin in close proximity with the NE, thereby contributing to the maintenance of the NE structure [5], [10]. LAP1 gained more attention when it was reported to interact with torsinA in the NE [11]. A mutation of a glutamic acid within torsinA is responsible for most cases of DYT1 dystonia, a neurological and movement disorder [12]. Thus, LAP1 is also known as torsinA interacting protein 1 (TOR1AIP1) and the gene encoding LAP1 is termed *TOR1AIP1*. More recently, LAP1 was found to interact with the INM protein emerin [13], which is associated with the X-linked Emery-Dreifuss muscular dystrophy disorder [14]. Furthermore, it was reported that conditional deletion of LAP1 from mouse striated muscle causes muscular dystrophy leading to early lethality [13]. We have recently reported that human LAP1B binds to protein phosphatase 1 (PP1) in the nucleoplasm and also that it is dephosphorylated *in vitro* by this phosphatase [15].

In the present study, we took advantage of the shRNA technology to knockdown LAP1 in human cells, so as to determine whether other human LAP1 isoform exist. Subsequently two isoforms, LAP1B and LAP1C, were identified. Using HPLC-mass spectrometry (MS) analysis, we showed that human LAP1C is putatively N-terminal truncated. The existence of this novel isoform LAP1C was confirmed by expressing HA-tagged LAP1C in human cells. LAP1C has never previously been identified in human cells, thus this is the first time that two human LAP1 isoforms have been described in human cells. Furthermore, the relative abundance of LAP1 isoforms in human cell lines was estimated. Finally, our data provided evidence that PP1 is responsible for dephosphorylating both Ser306 and Ser310 residues of LAP1B/LAP1C.

## Materials and Methods

### Antibodies

The primary antibodies used were rabbit polyclonal LAP1 [11]; rabbit polyclonal lamin B1 (Santa Cruz Biotechnology); mouse monoclonal β-tubulin (Invitrogen); mouse monoclonal synaptophysin (Synaptic Systems); rabbit polyclonal CBC3C that recognizes the C-terminal of PP1γ [16]; Myc-tag antibody (Cell Signaling), that recognizes Myc-fusion proteins; and HA-tag antibody (Clontech), that recognizes HA-fusion proteins. The secondary antibodies used were anti-mouse and anti-rabbit horseradish peroxidase-linked antibodies (GE Healthcare) for ECL detection.

### Expression vectors and DNA constructs

Myc-LAP1B and pET-LAP1B constructs have been previously described [15]. The pSIREN-RetroQ vector (Clontech) was kindly provided by Dr. Celso Cunha from the *Instituto de Higiene e Medicina Tropical*, Lisbon [17]. LAP1C was prepared by PCR amplification using the following primers: 5′-GAATTCATATGAAGACGCGAAGGAC-3′ and 5′-CTCGAGTTATAAGCAGATGCCCCT-3. The amplified fragment was subcloned into the *EcoRI/XhoI* restriction sites of the pCMV-HA vector (Clontech) to obtain a HA-fusion protein.

### Brain dissection

Winstar rats (9–12 weeks) were obtained from Harlan Interfaune Ibérica, SL. All experimental procedures observed the European legislation for animal experimentation (2010/63/EU). No specific ethics approval under EU guidelines was required for this project, since the rats were only euthanized, by cervical stretching followed by decapitation, for brain removal. This is within the European law (Council Directive 86/609/EEC) and during this procedure we took all steps to ameliorate animal suffering and used the minimum number of animals possible. The procedures were approved and supervised by our Institutional Animal Care and Use Committee (IACUC): Comissão Responsável pela Experimentação e Bem-Estar Animal (CREBEA). Animals were sacrificed by cervical stretching followed by decapitation, and the cortex was dissected out on ice. The tissue was then homogenized on ice, in lysis buffer (50 mM Tris-HCl pH 8.0, 120 mM NaCl, 4% CHAPS) containing protease inhibitors (1 mM PMSF, 10 mM Benzamidine, 2 µM Leupeptin, 1.5 µM Aprotinin, 5 µM Pepstatin A), with a Potter-Elvehjem tissue homogenizer with 10–15 pulses at 650–750 rpm [18].

### Cell culture and transfection

SH-SY5Y cells (ATCC CRL-2266) were grown in Minimal Essential Medium (MEM) supplemented with F-12 Nutrient Mixture (Gibco, Invitrogen), 10% fetal bovine serum (FBS, Gibco, Invitrogen), 1.5 mM L-glutamine and 100 U/mL penicillin, 100 µg/mL streptomycin and 0.25 µg/mL amphotericin B (Gibco, Invitrogen). In order to promote SH-SY5Y cells differentiation, cells were plated at a density of 1×10^5^ and grown for 10 days in MEM/F12 medium with 10% FBS in the presence of 10 µM retinoic acid [19]. HeLa cells (ATTC CRM-CCL-2) were grown in MEM with Earle's salts and GlutaMAX (Gibco, Invitrogen), supplemented with 10% FBS, 1% Non-Essential amino acids (Gibco, Invitrogen) and 100 U/mL penicillin, 100 µg/mL streptomycin and 0.25 µg/mL amphotericin B (Gibco, Invitrogen). SKMEL-28 cells were handled as previously described [20]. PC12 cells (ATCC CRL-1721) were cultured in RPMI1640 medium (Alfagene) supplemented with 5% FBS, 10% horse serum (Gibco, Invitrogen) and 100 U/mL penicillin, 100 µg/mL streptomycin and 0.25 µg/mL amphotericin B (Gibco, Invitrogen). These cells were plated onto poly-l-ornithine coated dishes [21]. All cultures were maintained at 37°C and 5% CO_2_.

Rat cortical primary cultures were established from embryonic day 18 embryos as previously described [22]. Briefly, after dissociation with 0.45 mg/ml trypsin, cells were plated onto poly-D-lysine-coated dishes at a density of 1.0×10^5^ cells/cm^2^ in B27-supplemented Neurobasal medium (Gibco, Invitrogen), a serum-free medium combination [23]. The medium was supplemented with glutamine (0.5 mM) and gentamicin (60 µg/ml). Cultures were maintained in an atmosphere of 5% CO_2_ at 37°C until 14 days *in vitro* (DIV) before being used for experimental procedures.

Transient transfections of SH-SY5Y cells were performed using TurboFect (Thermo Scientific) according to the manufacturer's protocols. After 24 hours of transfection, cells were harvested for experimental procedures.

### LAP1B knockdown

The knockdown of endogenous LAP1 in SH-SY5Y cells was achieved using a short hairpin RNA (shRNA) strategy. To construct shRNA-expressing vectors, oligonucleotides targeting the human LAP1B mRNA and the corresponding complementary sequences, were inserted into the pSIREN-RetroQ vector. The oligonucleotide sequences were designed using the online designer tool of *Clontech*, available at http://bioinfo.clontech.com/rnaidesigner. Two pairs of oligonucleotides were chosen: one aligning between exon 7 and 8 (5′-gatccGTGCTAAGCTCAGGATATCTTCAAGAGAGATATCCTGAGCTTAGCACTTTTTTACGCGTg-3′ and 5′-aattcACGCGTAAAAAAGTGCTAAGCTCAGGATATC′ TCTCTTGAAGATATCCTGAGCTTAGCACg-3′) and other in exon 10 (5′- gatccGGAAGAGACACTTGGAACATTCAAGAGATGTTCCAAGTGTCTCTTCCTTTTTTACGCGTg-3′ and 5′- aattcACGCGTAAAAAAGGAAGAGACACTTGGAACA TCTCTTGAATGTTCCAAGTGTCTCTTCCg-3′) of the LAP1 mRNA (NM_001267578). The underlined sequences denote the LAP1 shRNA sequence targeting in the LAP1 mRNA. A control shRNA was also generated, by using a negative control oligonucleotide that does not target any human transcript (5′-gatccGCTTCATAAGGCGCATAGCTATTCAAGAGATAGCTATGCGCCTTATGCTTTTTTg-3′). The oligonucleotides were annealed and subcloned into the *BamH*I and *EcoR*I sites of the pSIREN-RetroQ vector. The generated constructs pSIREN-LAP1-C1 (targeted to exon 7/8), pSIREN-LAP1-C2 (targeted to exon 10) and pSIREN-CMS (control) were verified by restriction analysis and DNA sequencing using an ABI PRISM 310 Genetic Analyzer (Applied Biosystems, Porto, Portugal). Constructs were then transfected using the TurboFect reagent (Thermo Scientific) according to the manufacturer's protocols.

### RT-PCR and sequencing

Adult brain poly A+ RNA (Clontech) was reverse transcribed using the SuperScript First Strand Synthesis System (Invitrogen) and the *TOR1AIP1* gene specific primer E10RV (5′-CTTGGCCTGACCTACTCTTAAGAC-3′) or the oligo(dT)_20_ primer. The synthetized cDNA was amplified using the following primer pairs: forward primer E1FW (5′-CAGGAGAACCTAGGGTCCATAAAG-3′) and reverse primer E10BRV (5′- GTGAACAATTCTCAGAACTTGGGAC-3′); forward primer E2FW (5′-CATTCC TCTGAAGAGGATG-3′) and reverse primer E10BRV; forward primer E5FW (5′ CTGAAGAAGATGATCAAGACAGCTC-3′) and reverse primer E10CRV (5′-GTGAGCAGTAAGATAGCAGGCTG-3′). The PCR products were excised from agarose gel and purified using QIAquick Gel Extraction Kit (Promega). The purified fragments were cloned into the Nzy-blunt PCR cloning kit (Nzytech). One clone from each reaction was selected and the inserts sequenced using an ABI PRISM 310 Genetic Analyzer (Applied Biosystems, Porto, Portugal).

### RNA isolation

Total RNA was isolated from SH-SY5Y cells using Trifast reagent (Peqlab Biotechnologie GmbH) following the supplier's protocols. Briefly, cells were homogenised in 500 µl of Trifast reagent with a 20 G needle. Then, cell lysates were incubated at room temperature for 5 min before 100 µL of chloroform were added. Samples were shaken vigorously for 15 seconds and kept at room temperature for 5 min before centrifugation at 12000 g for 15 min. The upper aqueous phase, containing RNA, was transferred into a new tube and the RNA precipitated by adding 250 µl of isopropanol followed by incubation at 4°C for 10 minutes. The RNA was spun down for 10 min at 12000 g and the pellet washed twice with 500 µl of ice cold 75% ethanol and subsequently centrifuged for 10 min at 12000 g. The RNA was air-dried at room temperature and dissolved in RNAse free water.

### Northern blot analysis

Northern blot analysis was performed following the protocol provided with the Odyssey Infrared Imaging System (LI-COR Biosciences) with some alterations. Briefly, 20 µg of total RNA from SH-SY5Y cell were mixed with formaldehyde/formamide RNA loading buffer. The samples were incubated at 65°C for 15 minutes, chilled on ice followed by the addition of loading buffer (50% glycerol, 1 mM EDTA pH 8.0, 0.25% bromophenol blue and 0.25% xylene cyanol FF). Then, the samples were loaded on 1% agarose gel in formaldehyde gel running buffer (0.02 M MOPS pH 7.0, 8 mM sodium acetate, 1 mM EDTA pH 8.0 and 2.2 M formaldehyde). Samples were transferred onto nitrocellulose membranes by capillary elution in 20× SSC buffer overnight. RNA was cross-linked to the membrane using a UV cross-linker. Membranes were hybridized with a LAP1 biotinylated probe. To generate the biotinylated probe, clone 1A (from the RT-PCR reaction E1FW + E10BRV) was used as a template for PCR amplification in the presence of biotin-16-dUTP (Roche) and the primers E10FW (5′-CAAGGTCAAGATGAGAAGCTG-3)′ and E10BRV (5′- GTGAACAATTCTCAGAACTTGGGAC-3′), both targeting exon 10 of LAP1. A probe directed to human β-actin was also generated using cDNA obtained by RT-PCR from human brain poly A+ RNA, and the primers AFW (5′-GCTCGTCGTCGACAACGGCT C-3′) and ARV (5′-CAAACATGAT CTGGGTCATCTTCTC-3′). The membrane was pre-hybridized at 42°C with ULTRAhyp-Oligo Buffer (Ambion) and hybridized overnight at 42°C with biotinylated probes (diluted in ULTRAhyb-Oligo Buffer) and then washed with Low and High Stringency Washes (Ambion). Membranes were blocked for 1 hour with Odyssey Blocking Buffer plus 1% SDS (LI-COR Biosciences) and then incubated for 1 hour with Streptavidin IRDye800 CW conjugate (1∶10000) in blocking buffer plus 1% SDS (LI-COR Biosciences). Blots were washed three times with PBS-T 1× and one with PBS 1× before scanning on an Odyssey IR Imaging System (LI-COR Biosciences).

### Co-immunoprecipitation

SH-SY5Y cells were collected in lysis buffer (50 mM Tris-HCl pH 8, 120 mM NaCl, 4% CHAPS) containing protease inhibitors (1 mM PMSF, 10 mM Benzamidine, 2 µM Leupeptin, 1.5 µM Aprotinin, 5 µM Pepstatin A). Dynabeads Protein G (Dynal, Invitrogen) were washed in 3% BSA/1× PBS. LAP1 antibody was cross-linked to Dynabeads according to the manufacturer's protocol. Cell lysates were precleared with 20 µL (0.6 mg) Dynabeads for 1 hour and then incubated with antibody-dynabeads overnight at 4°C. The immunoprecipitates were washed in 1× PBS and proteins eluted by boiling in loading buffer before SDS-PAGE.

### LAP1 solubilization assay

For the LAP1 solubilization we performed a procedure adapted from Goodchild, 2005 [11]. SH-SY5Y cells were collected in the following buffers supplemented with protease inhibitors (1 mM PMSF, 2 µM Leupeptin, 1.5 µM Aprotinin): 50 mM Tris-HCl pH 7.5; 50 mM Tris-HCl pH 7.5 and 1% triton X-100; 50 mM Tris-HCl pH 7.5, 1% triton X-100 and 50 mM NaCl or 500 mM NaCl. Homogenates were incubated on ice for 20 minutes and centrifuged at 20000 g for 15 min to separate supernatant (soluble) and pellet (insoluble) fractions. Pellet fractions were solubilized in 150 µl of 1× loading buffer at 90°C and supernatants were brought to a 150 µl volume and 1× concentration by adding 4× loading buffer. Equal volumes of pellet and supernatant were loaded on SDS-PAGE.

### Nano-HPLC and Mass spectrometry

For nano-HPLC-MS analysis, SH-SY5Y total cell lysates, SH-SY5Y cells membrane-containing fraction (pellet collected by centrifugation in 50 mM Tris-HCl pH 7.5, as previously described) and LAP1 immunoprecipitates (obtained as previously described) were resolved by 10% SDS-PAGE. Gels were stained with Coomassie blue colloidal (Sigma-Aldrich) using standard procedures [24]. Two bands were excised from the gel: one below 75 kDa and other above 50 kDa, corresponding to the molecular weights of LAP1B (68 kDa) and the putative LAP1C (56 kDa) protein, respectively. Bands were then excised from the gel and destained. The measurements were performed on a nano-HPLC system Ultimate 3000 (Dionex, Germany), coupled on-line to a quadrupole-orbitrap mass spectrometer (Q Exactive; Thermo Fischer Scientific, Germany). Samples were loaded on a C18 trap column (Thermo, 100 µm×2 cm, particle size 5 µm, pore size 100 Å, C18) with 0.1% TFA and 30 µl/min flow rate. Then the trap column was online switched with an analytical C18 column (Thermo, 75 µm×50 cm, particle size 2 µm, pore size 100 Å). The separation was performed with a flow rate of 400 nl/min using the following solvent system: (A) 0.1% FA; (B) 84% ACN, 0.1% FA. The separation gradient was from 4–40% B in 95 min, followed by a washing step at 95% B (5 min) and an equilibration step at 4% B (5 min). After nano-HPLC separation the eluent was ionized by a nano electrospray ionization source (ESI) and analyzed in data dependent scan mode in a Q Exactive. In the ESI/MS-MS analysis full MS spectra were scanned between 350–1400 m/z with a resolution of 70,000 at 200 m/z (AGC target 3e6, 80 ms maximum injection time). The capillary temperature was set at 250°C and the spray voltage at 1600 V (+). Lock mass polydimethylcyclosiloxane (m/z 445.120) was used for internal recalibration. The m/z values initiating MS/MS were set on a dynamic exclusion list for 30 s and the 10 most intensive ions (charge +2, +3, +4) were selected for fragmentation. MS/MS fragments were generated by higher energy collision induced dissociation (HCD) and the fragmentation was performed with 27% normalized collision energy (NCE). The first MS/MS mass was fixed at 130.0 m/z and isolation window 2.2 m/z. The fragment analysis was performed in an orbitrap analyzer with 35,000 resolution at 200 m/z (AGC 1e6, maximum injection time 120 ms).

### SDS-PAGE and immunoblotting

Samples were separated on SDS-PAGE and electrophoretically transferred onto nitrocellulose. Nitrocellulose membranes were incubated in Ponceau S solution for 5 minutes and then scanned in a GS-800 calibrated imaging densitometer (Bio-Rad), in order to assess equal gel loading. Membranes were washed in TBST 1× to remove Ponceau S staining, followed by immunological detection with specific antibodies as indicated. Membranes were saturated in 5% non-fat dry milk and further incubated with primary antibodies. Detection was achieved using horseradish peroxidase-conjugated anti-rabbit or anti-mouse IgGs as secondary antibodies and proteins visualized by ECL system (GE Healthcare).

### Immunocytochemistry

SH-SY5Y cells were fixed using 4% paraformaldehyde and further permeabilized with triton X-100 for 10 minutes, washed with PBS 1× and then blocked in 3% BSA/PBS 1× for 1 hour. Cells were incubated with the primary antibody against HA tag for 2 hours, followed by secondary antibodies for 1 hour. Preparations were washed with PBS, mounted using Vectashield mounting media with DAPI (Vector). Preparations were visualized using an LSM510-Meta confocal microscope (Zeiss) and a 63x/1.4 oil immersion objective. Microphotographs were acquired in a sole section in the Z-axis (xy mode) and represent a mean of 16 scans. The analysis was performed as previously described [18], [25].

### Bioinformatics analysis

Database searches to find homologies between sequences were performed using BLAST algorithm (http://blast.ncbi.nlm.nih.gov/Blast.cgi) [26]. Multiple sequence alignments were performed using the CLUSTAL OMEGA alignment tool (http://www.ebi.ac.uk/Tools/msa/clustalo/) [27]. The splice prediction was achieved by using the programs NNSPLICE (http://www.fruitfly.org/seq_tools/splice.html) and GENSCAN (http://genes.mit.edu/GENSCAN.html) [28], [29]. The prediction of promoter and transcription factor binding sites was performed using the NNPP program (http://www.fruitfly.org/seq_tools/promoter.html) [30] and the TSSG (http://linux1.softberry.com/berry.phtml) [31].

### Quantification and Statistical Analysis

Autoradiograms were scanned in a GS-800 calibrated imaging densitometer (Bio-Rad) and protein bands quantified using the Quantity One densitometry software (Bio-Rad). Data were expressed as mean ± SEM of at least three independent experiments. Statistical significance analysis was conducted by Student's test, with the level of statistical significance being considered P<0.05.

## Results

### Knockdown of human LAP1

To date little information is available regarding the human LAP1 family of proteins and their physiological functions. Recently, we described that one of the family members, LAP1B, is a novel PP1 binding protein [15]. To clarify whether additional human LAP1 family members exist and their physiological impact, we generated LAP1 specific shRNAs to reduce the cellular levels of LAP1 protein [32]. For this purpose, a pSIREN-RetroQ vector coding for LAP1-specific shRNAs: pSIREN-LAP1-C1 and pSIREN-LAP1-C2 were designed to align between exons 7/8 and in exon 10 of LAP1, respectively. SH-SY5Y cells were transfected with one of the pSIREN-LAP1 plasmids or with both for 24 hours. In parallel, SH-SY5Y cells were also transfected with the negative control, the pSIREN-CMS construct. The efficiency of LAP1 knockdown was monitored by immunoblotting with a LAP1 specific antibody [11] in the cell lysates resulting from the above mentioned experiments. This LAP1 antibody was raised against residues 463–478 (exon 10) of mouse LAP1 and is able to detect the three LAP1 splice variants in mouse cells [11]. Given that the amino acid identity between mouse and human LAP1 is very high in the region recognized by this antibody, the same antibody was used to detect human LAP1.

**Figure 1:**
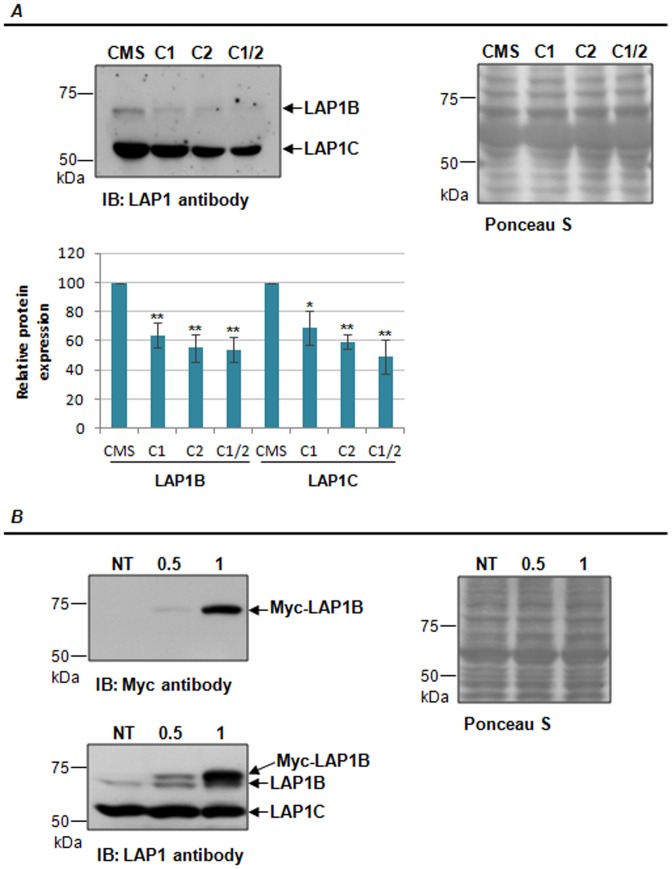
Identification of a new putative human LAP1 isoform. **A-**Transfection of SH-SY5Y cells with pSIREN-RetroQ vector coding for LAP1-specific shRNAs resulted in the knockdown of two LAP1 isoforms: LAP1B and putative LAP1C. Data are presented as mean ± SEM of at least three independent experiments. Statistically different from CMS transfected cells, *p<0.05, **p<0.01. C1, pSIREN-C1 (directed against exon 7/8 of LAP1), C2, pSIREN-C2 (directed against exon 10 of LAP1); CMS, pSIREN-CMS (control missense). **B-** Transfection of SH-SY5Y cells with Myc-LAP1B (0.5 or 1 µg). Ponceau S staining was used as loading control. NT, non-transfected; IB, immunoblotting.

Two major peptides, with reduced endogenous LAP1 levels in cell lysates, were detected upon transfecting with the pSIREN-LAP1-C1, pSIREN-LAP1-C2 or both constructs simultaneously (Figure 1A). The higher migrating band (approximately 68 kDa) corresponds to the molecular weight of the known LAP1B isoform, while the lower band (approximately 56 kDa) had not been previously reported in human cells, but has the same molecular weight as that of rat LAP1C, described in the literature. Therefore we hypothesized that this novel immunoreactive band is likely to correspond to the human LAP1C isoform. The intracellular levels of LAP1B were reduced by 34%, 45% and 47% upon transfection with pSIREN-LAP1-C1, pSIREN-LAP1-C2 or both constructs, respectively (Figure 1A). In a similar fashion the intracellular levels of the putative LAP1C were also reduced by 31%, 41% and 51%, upon transfection with pSIREN-LAP1-C1, pSIREN-LAP1-C2 or both constructs together, respectively. Ponceau S staining (Figure 1A) was used as loading control as previously described [33], [34]. The response obtained also permits to conclude that both LAP1B and the putative newly described human isoform, here designated LAP1C, have in common the regions of exon 7, 8 and 10 targeted by the shRNAs used, which corroborates the fact that all LAP1 isoforms have a conserved C-terminal.

In order to clarify that the new putative human LAP1C isoform is not a product of cleavage or post-translational proteolytic processing of LAP1B, we transfected SH-SY5Y cells with two different amounts of Myc-LAP1B (0.5 µg and 1 µg). After immunoblotting with Myc antibody, only one band was detected corresponding to the transfected Myc-LAP1B (Figure 1B). Moreover, we performed immunoblotting with LAP1 antibody and did not detect an increase in the expression of the endogenous putative LAP1C immunoreactive band upon transfection (Figure 1B). Ponceau S staining (Figure 1B) was used to confirm that equal amount of protein was loaded in each well. These results support the fact that the putative LAP1C is not a product of LAP1B cleavage or proteolytic processing, but in fact a distinct isoform.

### *In silico* analysis of the *TOR1AIP1* genes

**Figure 2:**
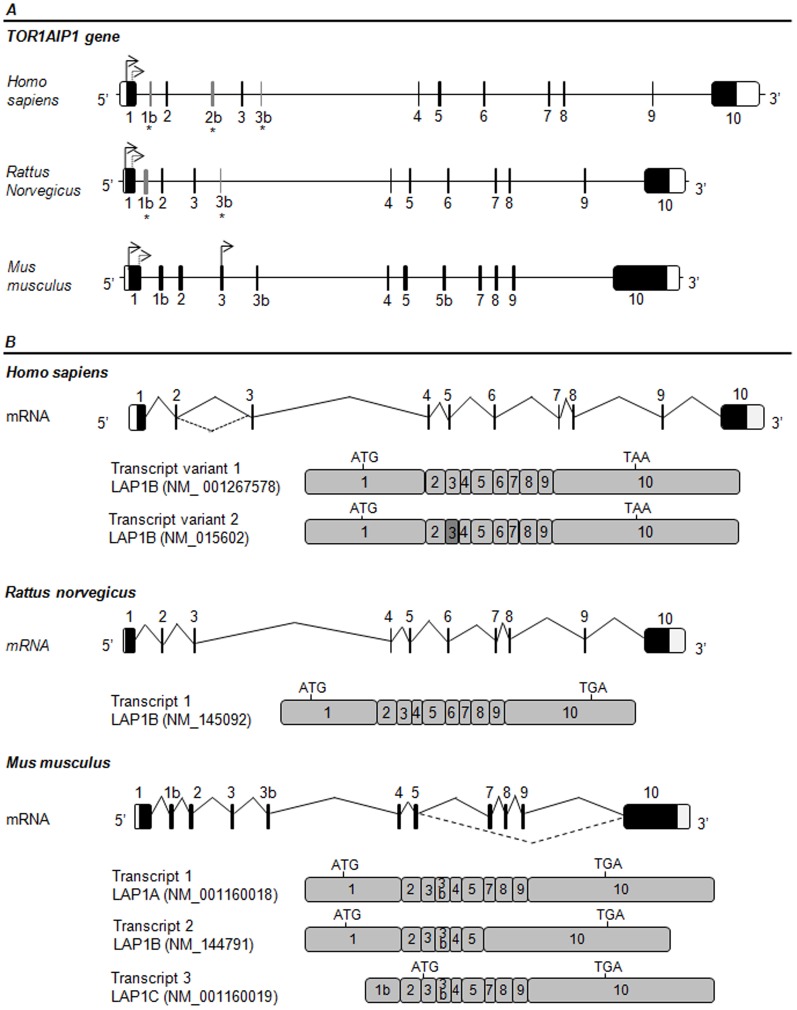
Gene structure and splice variants of human, rat and mouse *TOR1AIP1*. **A-** Structure of the human, rat and mouse *TOR1AIP1* gene (coding for LAP1). Non-coding sequences in exons are represented by open boxes and coding exons are represented by black filled boxes. Predicted exons 1b and 3b in human and rat genes, and exon 2b in human gene are represented by grey boxes and denoted by an asterisk. The in-frame ATG codons are indicated by arrows. **B-** Schematic representation of the alternative splicing patterns and resulting LAP1 transcripts variants (human, rat and mouse). The translation initiation codons (ATG) and the stop codons (TAA or TGA in human and mouse/rat sequences, respectively) are indicated in each transcript. Human LAP1 transcripts variants differ only in exon 3 (dark grey) by three nucleotides through an alternative 3′ splicing event.

*In silico* analysis of the *TOR1AIP1* gene was performed to address the potential diversity of human LAP1 proteins. Two human LAP1 transcripts have in fact been reported [7], [8]. Bioinformatic analysis of those transcripts and the alignment with the genomic sequence, revealed the presence of 10 exons (Figure 2A). Transcript variant 1 [GenBank: NM_001267578] represents the longest transcript and is identical to the first human LAP1B sequence reported in 2002 [6]. This transcript differs from variant 2 [GenBank: NM_015602], only by a CAG insertion, which results in an additional alanine in the coding sequence (Figure 2B). Some reports showed that *TOR1AIP1* gene possesses a 3′ tandem splice site, TAGCAG, at the exon 3 boundary, which results in one amino acid insertion or deletion in the encoded protein [7], [8].

Sequencing of rat LAP1C and partial characterization of rat LAP1A and LAP1B suggested that rat LAP1 family members arise from alternative splicing [5]. However, despite what is reported in the literature, only one Reference Sequence (RefSeq) transcript in GenBank was found that corresponds to rat LAP1B isoform (Figure 2B) [GenBank:NM_145092]. Nevertheless, two related sequences (non-RefSeq sequences) were found in GenBank: U20286 (transcript 2), a transcript that lacks an N-terminal segment and U19614 (transcript 3), a transcript that lacks an internal segment (Figure S1). Alignment of the rat LAP1 genomic sequence with the known rat LAP1B transcript, using the BLAST algorithm, revealed the presence of 10 exons (Figure 2). Taking into account the exon structure of rat LAP1 transcripts, we infer that U20286 has a truncated exon 1 in the N-terminal, while in the U19614 transcript, exon 5 was skipped.

For mouse there are three RefSeq records corresponding to three different mouse LAP1 transcripts: transcript 1 [GenBank: NM_001160018] that represents the longest transcript; transcript 2 [GenBank: NM _144791] that is shorter than transcript 1 and lacks an internal segment; and transcript 3 [GenBank: NM_001160019] that represents the smallest transcript and lacks the N-terminus (Figure 2B). Additionally, we found other related sequences corresponding to two different mouse LAP1 transcripts in GenBank: AK152751 (transcript 4), a transcript that lacks an N-terminal segment and AB251963 (transcript 5), a transcript that has an additional internal segment (Figure S1). Alignment of the mouse LAP1 genomic sequence with the known transcripts revealed the presence of 12 exons (Figure 2). Taking into account the exon structure of mouse LAP1 transcripts, we showed that exon 7, 8 and 9 are absent in transcript 2. Transcript 3 lacks exon 1, but has an additional first exon, that we termed exon 1b. However translation is not initiated at the exon 1b, but exon 3 does have an in frame ATG, encoding for a protein with a different N-terminal. Transcript 4 has a truncated exon 1 in the N-terminal and transcript 5 has an alternative exon 5b that is not found in any of the other transcripts. Of note, the C-terminal (exon 10) seems to be the most conserved region among mouse LAP1 isoforms.

In order to predict alternative exons, which would lead to distinct human LAP1 isoforms, we aligned mouse LAP1 transcripts against the genomic sequence of the *TOR1AIP1 gene*, using BLAST algorithm. Further, we identified intron-exon junctions by comparing genomic and cDNA sequences and making use of *in silico* tools NNSPLICE and GENSCAN (Table S1). The alignment revealed high identity between exons 1–5 and 7–10, while exon 6 of human gene does not align with any mouse transcript. Moreover, mouse transcripts have three different exons (that we termed 1b, 3b and 5b) that are not found in the human LAP1B transcripts. The alignment of mouse exons 1b and 3b against the human genomic sequence revealed the presence of putative alternative exons 1b and 3b in the human gene (Figure 2A). In the GenBank database, a human expressed sequence tag (EST) was found [GenBank: DB454036], which shares homology with exon 1b. Alignment of this EST with human genomic sequence also revealed the presence of an alternative exon, that we termed exon 2b (Figure 2A). This exon is not conserved in mouse or rat genomic sequences (Table S1). In contrast, a human EST [GenBank: CX760895] encoding an exon with high homology with mouse exon 3b was found in the GenBank database. The splice sites of putative exon 2b and 3b are according to the consensus rules (GT/AG).

Additionally, searches in Genbank database for human ESTs and alignments of detected human ESTs against the genomic sequence of human LAP1 were performed, in order to identify alternative exons. It was found that the 3′ end of exon 8, exon 9 and the 5′ end of exon 10 were skipped in one EST [GenBank: AU154882]. Furthermore, a non-RefSeq protein record [GenBank: EAW91067] that matches with this EST was found. Finally, several human ESTs (e.g. AL530866, BM907999, DA552747 and DA539545) and non-RefSeq mRNAs records (BC023247, AK023204 and AK021613) that lack the 5′ end region of exon 1 were found in GenBank. These sequences lack the start codon present in LAP1B but have an additional in frame ATG, 363 nucleotides downstream of the LAP1B start codon (Figure 2A). This second in frame ATG is conserved between species (human, mouse and rat). Once more the analysis of the identified transcripts reveals that the C-terminal region of LAP1 transcripts remains highly conserved.

### Analysis of LAP1 transcripts

#### RT-PCR

**Figure 3:**
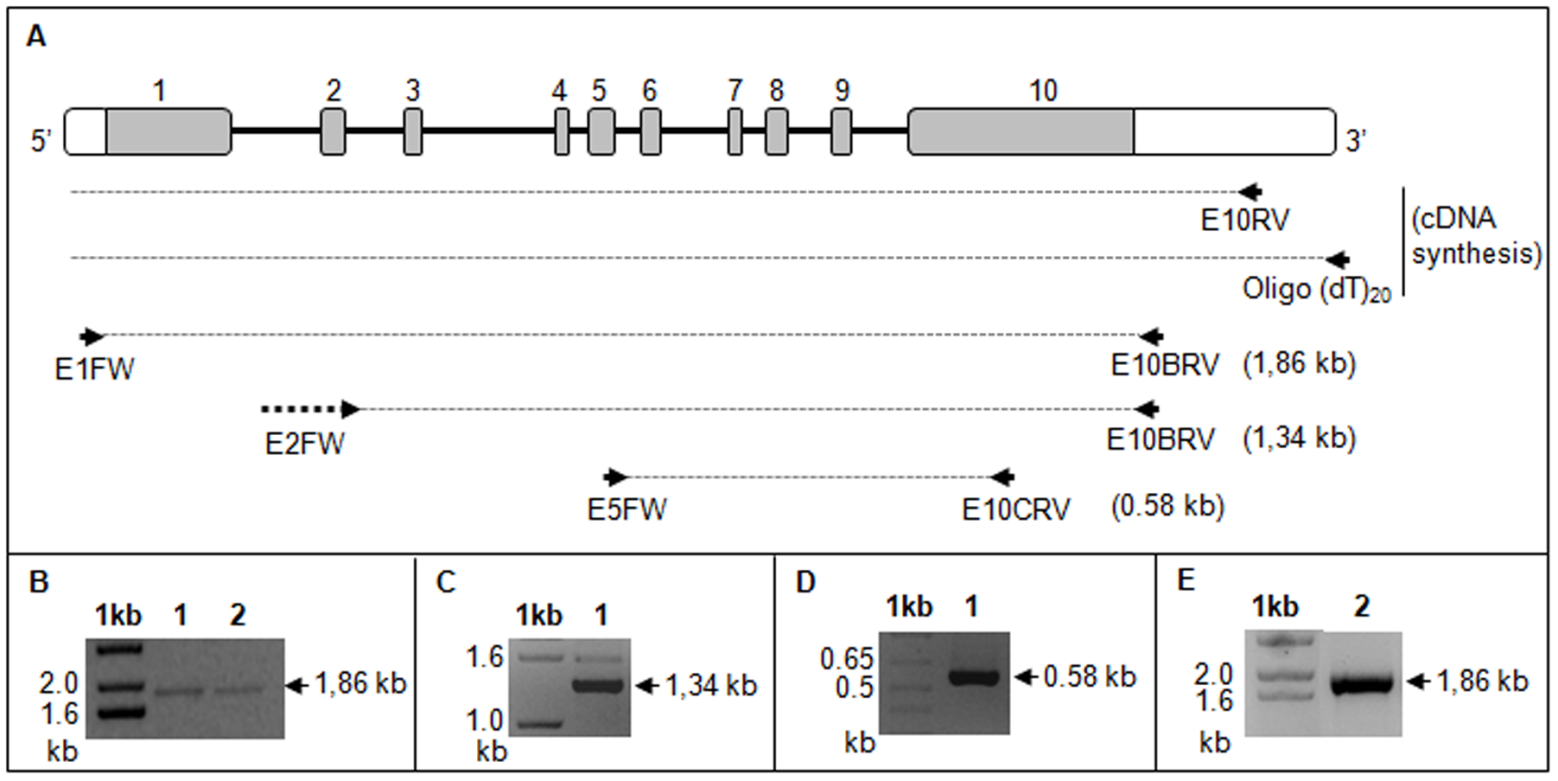
RT-PCR of human LAP1. **A-** Localization of the primers used for RT-PCR on human *TOR1AIP1* gene. The cDNA was synthesized from adult brain poly A+ RNA (Clontech) (B, C and D) or SH-SY5Y cell total RNA (E) using a reverse primer targeted to exon 10 (E10RV) or an oligo(dT)_20_ primer. Amplification of the cDNA was performed using specific primer pairs. **B-** cDNA amplification using primers E1FW (targeted to exon 1) and E10BRV (targeted to exon 10). **C-** cDNA amplification using primers E2FW (targeted to exon 1/2) and E10BRV (targeted to exon 10). **D-** cDNA amplification using primers E5FW (targeted to exon 5) and E10CRV (targeted to the middle of exon 10). **E-** cDNA amplification using primers E1FW (targeted to exon 1) and E10BRV (targeted to exon 10). 1kb, DNA size marker 1kb ladder (Invitrogen); 1, cDNA synthesized using E10RV primer; 2, cDNA synthetized using oligo(dT)_20_ primer.

Given that two human LAP1 immunoreactive bands were detected (Figure 1) and since there is evidence supporting that LAP1 isoforms are originated by alternative splicing (Figure 2), we went on to test if the smaller LAP1 transcript (putative LAP1C) has alternative exons, using RT-PCR. Briefly, cDNA was synthetized from adult brain poly A+ RNA using a reverse primer in the 3′ UTR of exon 10 (E10RV) of human LAP1B or an oligo(dT)_20_ primer that aligned with the poly A+ tail of mRNAs (Figure 3A). Subsequent amplification reactions were carried out using different primer pairs. The primer set E1FW (targeted to exon 1) and E10BRV (targeted to exon 10) amplified a fragment of 1.86 Kb (Figure 3B). This PCR fragment was ligated into the Nzy-blunt vector and the insert fully sequenced. Sequence results showed that the PCR fragment corresponds to the LAP1B transcript already described, as expected by its size. Given that only one fragment was amplified, using primers that aligned in exon 1 and 10, one can deduce that the N- or the C-terminal of the putative LAP1C transcript should be different. Additionally, and in order to exclude the possibility of internal alternative exons, the cDNA was also amplified using the following primer sets: E2FW (targeted to the boundary between exon 1 and 2) and E10BRV (targeted to exon 10); E5FW (targeted to exon 5) and E10CRV (targeted to an internal region of exon 10). The first primer set (E2FW + E10BRV) used, generated a fragment of 1.34 kb (Figure 3C), which corresponds to the expected size of the transcript without alternative exons. Additionally, a larger fragment of 1.6 kb was also obtained which, after sequencing analysis, corresponded to an unspecific band. The second primer set (E5FW + E10CRV) used, generated a fragment of 0.58 kb (Figure 3D) that matched with the expected transcript without alternative exons. In addition, total RNA from SH-SY5Y cells was extracted and RT-PCR was performed using the oligo(dT)_20_ primer. The synthesized cDNA was amplified using the primers E1FW and E10BRV and once more a single PCR fragment of 1.86 kb was obtained, corresponding to the LAP1B transcript (Figure 3E).

#### Northern Blot

**Figure 4:**
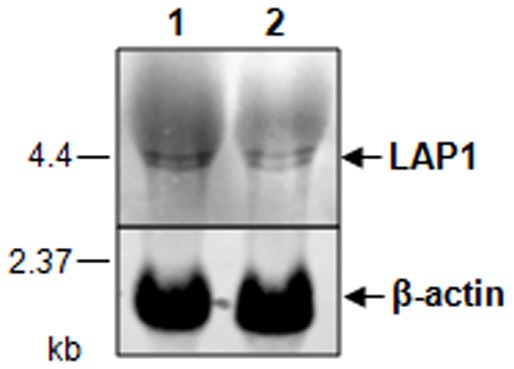
Northern blot analysis of LAP1 RNAs in SH-SY5Y cells. **A-** Total RNA was isolated from SH-SY5Y cells and membranes hybridized with a biotinylated probe directed against exon 10 of LAP1. β-actin was probed as control. RNA size markers are depicted on the left. 1, RNA isolated from SH-SY5Y cells non-differentiated; 2, RNA isolated from SH-SY5Y cells differentiated with retinoic acid.

The RT-PCR methodology did not produce a transcript corresponding to the putative LAP1C isoform, nor did it corroborate the presence of alternative exons that would lead to the translation of LAP1C. Consequently, in order to test whether different mRNAs or a single mRNA encodes LAP1 isoforms, Northern blot analysis was performed. If a single RNA is present, LAP1 isoforms could be generated by an alternative translation initiation mechanism, instead of alternative transcription. Hence, a probe was designed, directed against a region of exon 10 (span nucleotides 1602–2265) that is conserved in LAP1 isoforms. Total RNA from SH-SY5Y cells was isolated, given that this cell line expresses high levels of the putative LAP1C isoform (Figure 1B). Both undifferentiated and differentiated (with 10 µM retinoic acid for 4 days) SH-SY5Y cells were used to isolate total RNA. The results showed that the probe hybridized with two bands in both conditions (Figure 4). The higher band corresponds to the LAP1B transcript but appears to migrate slower than expected, bearing in mind its characterized mRNA size of 4.05 kb (GenBank: NM_001267578). The presence of a lower band is consistent with the existence of a second LAP1 transcript, corresponding to putative LAP1C transcript. A probe directed at human β-actin was used as a control and hybridized to a single band below 3.7 kb (Figure 4), as expected.

Furthermore, we showed that *in vitro* translation of LAP1B does not generate a low molecular weight protein, indicating that the putative LAP1C is not generated by alternative translational initiation (Figure S2).

### Identification of LAP1C isoform by liquid chromatography-mass spectrometry

Northern blot analysis supported the existence of two LAP1 isoforms in human cell lines, but data was not as clear from the other methodologies, as described above. Thus, HPLC-MS analysis was employed. Two approaches were used for enrichment of LAP1 peptides. In the first procedure, membrane proteins from SH-SY5Y cells were enriched by centrifugation in 50 mM Tris-HCl buffer (insoluble fraction, pellet) and in the second, SH-SY5Y cell lysates were immunoprecipitated with the LAP1 specific antibody. SH-SY5Y total cell lysates were also employed for HPLC-MS analysis. All three samples were loaded on SDS-PAGE followed by Coomassie blue colloidal staining (Figure S3). The bands including the LAP1B (68 kDa) and LAP1C (56 kDa) proteins were excised (Figure S3) and analyzed by HPLC-MS. Following careful excision, bands were tryptically digested, and the resulting peptides analysed in a nano-HPLC system online, coupled to a Q Exactive mass spectrometer (Thermo Fisher Scientific, Germany). Overall, 80 unique peptides of LAP1B/LAP1C were identified, for all the conditions analysed. Immunoprecipitation of LAP1 and isolation of membrane proteins showed to be efficient techniques for the enrichment of LAP1 isoforms, since a large number of peptides were identified in comparison with the number of peptides identified from total cell lysates (Table S2). After comparison of all peptides, 28 different peptides of LAP1B/LAP1C were identified. Overall, only 3 peptides were specifically identified in the 68 kDa band (containing LAP1B) and 11 peptides were only found in the 56 kDa (containing LAP1C) band. However, all these 11 peptides also match with the known sequence of LAP1B (Table 1). The overall sequence coverage was 47% for LAP1B and 75.3% for LAP1C. Since the LAP1C protein is more abundant in SH-SY5Y cells than LAP1B, it was expected that more peptides in the 56 kDa band (LAP1C) would be identified). Taking into account the exons boundaries of LAP1B represented in table 1, it is reasonable to conclude that the three unique peptides found in the LAP1B-containing bands correspond to the 5′ end region of exon 1. Moreover, we identified peptides that aligned fully or partially with the 10 exons of LAP1B (Table 1). Additionally, a putative methionine at position 122, which is a few amino acids upstream of the location of the first peptide identified in the LAP1C-containing bands (Table 1) was identified, indicating a potential start codon for LAP1C translation. This data is in agreement with GenBank AAH23247 sequence, in which the amino acid sequence was deduced by conceptual translation of the BC023247 mRNA sequence. When these values are compared with the expected molecular weight of LAP1 isoforms (Table 2), one can conclude that the band migrating at 56 kDa is consistent with the existence of a LAP1 isoform with a truncated exon 1 (LAP1C). Therefore, the newly identified LAP1C has a shorter exon 1 when compared to LAP1B, generating an putative N-terminal truncated protein. Given its molecular weight similarity to the rat LAP1C the authors propose that the new human LAP1 isoform should also have the designation LAP1C.

**Table 1:** LAP1B and LAP1C peptides identified by liquid chromatography-mass spectrometry.

	Peptides	Position of peptides in the known LAP1B sequence
Peptides identified in the 68 kDa band (LAP1B)	EGWGVYVTPR	
	LAPQNGGSSDAPAYR	
	FSDEPPEVYGDFEPLVAK	
	LQQQHSEQPPLQPSPVMTR	1 magdgrraea vr**egwgvyvtpr**apiregrg **rlapqnggss** **dapayr**tpps rqgrrevr**fs**
	DSHSSEEDEASSQTDLSQTISK	61 **deppevygdfeplvak**ersp vgkrtrleef rsdsakeevr esayylrsrq rrqprpqete
	DSHSSEEDEASSQTDLSQTISKK	121 emktrrttr**lqqqhseqpplqpspvmtrr**g lr**dshsseEd eassqtdlsqtiskk**tvr**si**
	SIQEAPVSEDLVIR	181 **qeapAvsedlvir**lrrpplr ypryEatsvq qk**vnfseeGeteeddqdsshssvttvk**ars
	VNFSEEGETEEDDQDSSHSSVTTVK	241 rdsdesgDkt trsssqyies fwqssqSqnf tahdk**qpsvlSsgyqk**tpqe wapqtarirt
	QPSVLSSGYQK	301 rmqNdsilks elgnqspsts sr**Qvtgqpqnasfvkr**nrww llpliaalas gsfwffstpe
	QVTGQPQNASFVK	361 vettavqefq nqmnqlknky qgqdeklwkr sqtflekhln sshpr**sqpaillltaar**dae
	QVTGQPQNASFVKR	421 ealrclseqi adayssfrsv rairidgtdk **atqdsdtvklevdqelsngfk**ngqnaavvh
	SQPAILLLTAAR	481 r**fesfpagstlifyk**ycdhe naafkdvalv ltvlleeetl gtslglkeve ekvrdflkvk
	ATQDSDTVKLEVDQELSNGFK	541 ftnsntpnsy nhmdpdklng lwsr**ishlvlpvqpenalkr** gicl
	LEVDQELSNGFK	
	FESFPAGSTLIFYK	
	ISHLVLPVQPENALK	
	ISHLVLPVQPENALKR	
Peptides identified in the 56 kDa band (LAP1C)	LQQQHSEQPPLQPSPVMTR	
	LQQQHSEQPPLQPSPVMTRR	
	DSHSSEEDEASSQTDLSQTISK	
	DSHSSEEDEASSQTDLSQTISKK	
	SIQEAPVSEDLVIR	
	RPPLRYPR	
	VNFSEEGETEEDDQDSSHSSVTTVK	1 magdgrraea vregwgvyvt prapiregrg rlapqnggss dapayrtpps rqgrrevrfs
	SSSQYIESFWQSSQSQNFTAHDK	61 deppevygdf eplvakersp vgkrtrleef rsdsakeevr esayylrsrq rrqprpqete
	QPSVLSSGYQK	121 e**M**ktrrttr**lqqqhseqppl qpspvmtrr**g lr**dshsseEd eassqtdlsq tiskk**tvr**si**
	TPQEWAPQTAR	181 **qeapAvsedl vir**lr**rpplr ypr**yEatsvq qk**vnfseeGe teeddqdssh ssvttvk**ars
	TRMQNDSILKSELGNQSPSTSSR	241 rdsdesgDkt tr**sssqyies fwqssqSqnf tahdkqpsvl Ssgyqktpqe wapqta**rir**t**
	MQNDSILKSELGNQSPSTSSR	301 **rmqNdsilks elgnqspsts srQvtgqpqn asfvkr**nrww llpliaalas gsfwffstpe
	QVTGQPQNASFVK	361 vettavqefq nqmnqlk**nky qgqdeklwk**r sqtflekhln sshpr**sqpai llltaardae**
	QVTGQPQNASFVKR	421 **ealr**clseqi adayssfrsv rair**idgtdk atqdsdtvkl evdqelsngf k**ngqnaavvh
	NKYQGQDEKLWK	481 r**fesfpagst lifyk**ycdhe naafk**dvalv ltvlleeetl gtslglk**eve ekvrdflkvk
	SQPAILLLTAAR	541 **ftnsntpnsy nhmdpdklng lwsrishlvl pvqpenalkr** gicl
	SQPAILLLTAARDAEEALR	
	IDGTDKATQDSDTVKLEVDQELSNGFK	
	ATQDSDTVKLEVDQELSNGFK	
	LEVDQELSNGFK	
	FESFPAGSTLIFYK	
	DVALVLTVLLEEETLGTSLGLK	
	FTNSNTPNSYNHMDPDKLNGLWSR	
	ISHLVLPVQPENALK	
	ISHLVLPVQPENALKR	

The peptides identified in the 68 kDa band (corresponding to LAP1B) and in the 56 kDa band (corresponding to LAP1C) are listed. The localization of those peptides (in bold) is shown in the amino acid sequence of the known LAP1B for comparative analysis. Amino acids located in exon boundaries are represented in upper case and underlined. A second methione, that may represent the translation start site of LAP1C, is representated in upper case (in bold) and underlined thickly.

**Table 2:** Human LAP1 transcripts and isoforms.

	Human LAP1 transcripts					
	LAP1B (variant 1)		LAP1B (variant 2)		LAP1C	
Exon	Total	Coding	Total	Coding	Total	Coding
**1**	936	475	693	475	NC	112
**2**	78	78	78	78	78	78
**3**	60	60	57	57	60	60
**4**	42	42	42	42	42	42
**5**	87	87	87	87	87	87
**6**	57	57	57	57	57	57
**7**	42	42	42	42	46	46
**8**	69	69	69	69	69	69
**9**	57	57	57	57	57	57
**10**	2624	785	2624	785	2624	785
**mRNA size (bp)**	4054	1752	4051	1749	>3358	1386

	Human LAP1 isoforms		
**N° of amino acids**	584	583	462
**Calculated MW (kDa)**	66.3	65.6	52.5
**Migration in SDS-PAGE (kDa)**	68	68	56

Organization and exon size (bp) of the previously described LAP1B transcripts and the new LAP1C transcript is described. The number of amino acids, calculated molecular weight (MW) and MW inferred through migration in SDS-PAGE gel of LAP1 isoforms are also shown. NC, not confirmed. The full size of exon 1 and the mRNA of LAP1C was not confirmed.

### Identification of a putative promoter in LAP1C sequence

**Figure 5:**
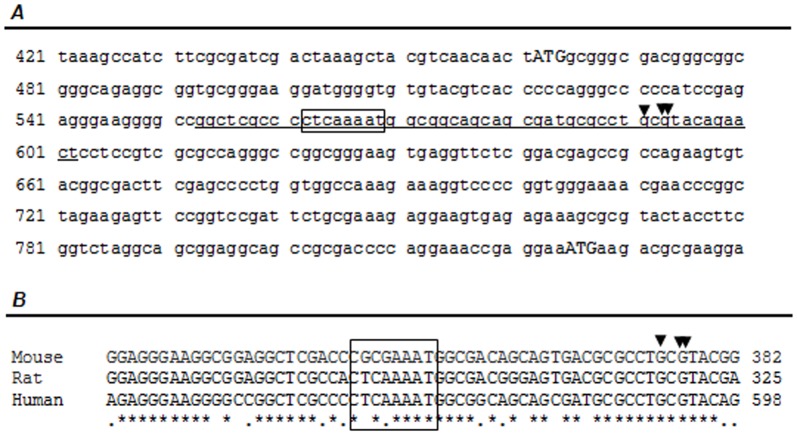
Predicted promoter and alternative transcription initiation site of human LAP1C. **A-** Localization of a predicted promoter in the *TOR1AIP1* genomic sequence. The promoter region predicted using the NNPP program is underlined. The transcription initiation site predicted by the TSSG program is indicated by an arrow and the one predicted by the NNPP program is indicated by a double arrow. The putative TATA box predicted using the TSSG program is indicated by a box. **B-** Alignment of the predicted human promoter region with the homologous mouse and rat sequences.

This study clearly demonstrates that two LAP1 isoforms are present in human cells and are most likely generated by the translation of two different mRNAs. Therefore, the regulatory mechanism underlying the expression of two LAP1 isoforms may be due to the transcriptional regulation of LAP1. Therefore, the genomic sequence of the first exon of the human *TOR1AIP1* gene was analyzed, in order to find putative alternative promoters that would lead to a downstream alternative transcription initiation and produce the smaller LAP1C transcript. NNPP and TSSG programs permitted the search for candidate promoters. These programs predicted a promoter in the region downstream of the translation initiation site of LAP1B and upstream of the translation initiation site of LAP1C (Figure 5A). A putative TATA box was predicted 29 nucleotides downstream from the predicted transcription initiation site, using the TSSG program. Furthermore, the region of the predicted transcription initiation site is conserved between species (Figure 5B).

### Characterization of the novel LAP1C isoform

**Figure 6:**
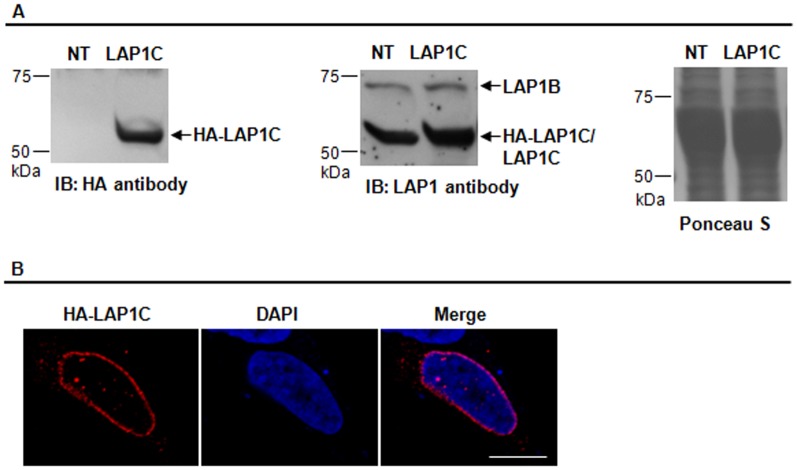
Expression and localization of HA-tagged LAP1C in human cells. SH-SY5Y cells were transfected with HA-LAP1C (LAP1C). **A-** Immunoblotting analysis using a HA antibody, which detected the transfected HA-LAP1C, or with a LAP1 antibody that detected endogenous LAP1 isoforms and transfected HA-LAP1C. Ponceau S staining was used to check equal loading. **B-** Immunolocalization of HA-LAP1C. Specific primary antibody against HA tag was detected with Alexa Fluor 594-conjugated secondary antibody (red). DNA was stained with DAPI NT, non-transfected; IB, immunoblotting.

The expected molecular weight of the LAP1C isoform is 52.5 kDa (Table 2) and the band detected using a specific LAP1 antibody is around 56 kDa (Figure 1 and Table 2). Therefore, in order to clearly establish that the 56 kDa band corresponds to the molecular weight of LAP1C, a LAP1C construct in fusion with an HA tag was generated and transfected into SH-SY5Y cells. The results showed that, after immunoblotting with HA antibody, a band of approximately 56 kDa was detected (Figure 6), which corresponds to the expected molecular weight of the transfected HA-LAP1C. Moreover the transfected HA-LAP1C co-migrates with the endogenous LAP1C and both are detected using a specific LAP1 antibody (Figure 6A). Equal amounts of protein were loaded in both lanes as confirmed by Ponceau S staining (Figure 6A) of the membrane. Additionally immunolocalization of the HA-LAP1C was determined in SH-SY5Y cells (Figure 6B). This result indicates that HA-LAP1C is mainly found in the nuclear envelope and also in some points inside the nucleus (Figure 6B).

### Additional functional characterization of LAP1 isoforms

#### Solubilization properties of LAP1 isoforms

**Figure 7:**
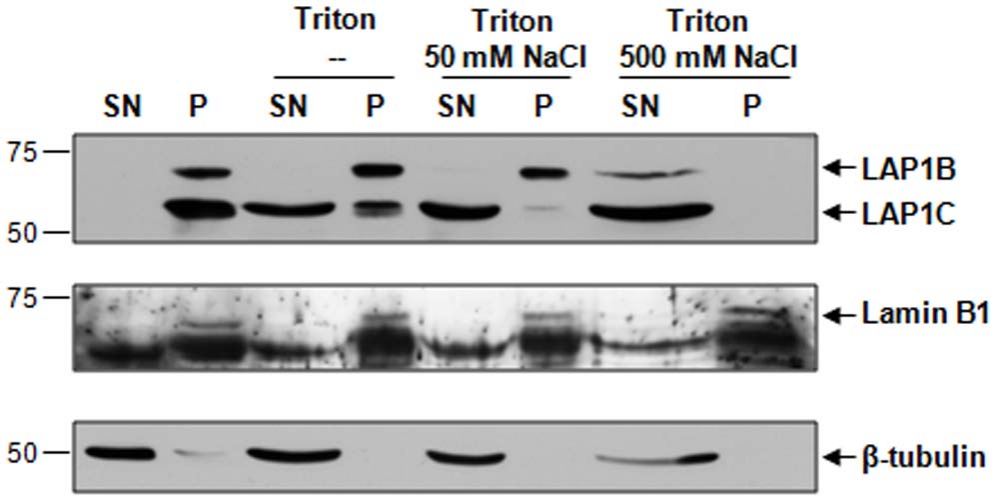
Solubilization properties of human LAP1 isoforms. Solubilization of LAP1 in Tris-HCl buffer or Tris-HCl buffer containing 1% triton X-100, 1% triton X-100 and 50 mM NaCl or 500 mM NaCl. Equal fractions of supernatant (SN) and pellet (P) were loaded. IB, immunoblotting.

Previous reports on the subcellular localization of different LAP1B deletion mutants demonstrated that only constructs with the whole nucleoplasmic domain were fully resistant to extraction with triton X-100. In contrast deletion mutants containing only a part of the nucleoplasmic domain were extractable using this detergent [6]. Furthermore, it was reported that most of the rat LAP1C is solubilized using triton X-100 plus 100 mM NaCl, while LAP1A and LAP1B remain in the pellet (insoluble fraction) along with the lamins [9]. Therefore, we went on to test if the human LAP1C isoform is less resistant to extraction from nuclear membranes using triton X-100, with increasing salt concentrations (NaCl). The results showed that LAP1C is partially solubilized after triton X-100 addition, while LAP1B remains in the pellet (Figure 7). Furthermore, the majority of LAP1C is solubilized after extraction with triton X-100 plus 50 mM NaCl and it is not found in the pellet using high salt concentration (500 mM). In contrast, LAP1B is only fully solubilized after extraction with triton X-100 plus 500 mM NaCl (Figure 7). Lamin B1 and β-tubulin were used as controls. As expected, lamin B1 is found in the pellet fraction while β-tubulin is found in the supernatant for all conditions tested (Figure 7). There is just a minor amount of β-tubulin in the pellet fraction when neither triton nor NaCl are added. These results are in agreement with the fact that human LAP1C differs from LAP1B in the first exon located in the nucleoplasmic domain.

#### Cell and tissue specific expression pattern of LAP1 isoforms

**Figure 8:**
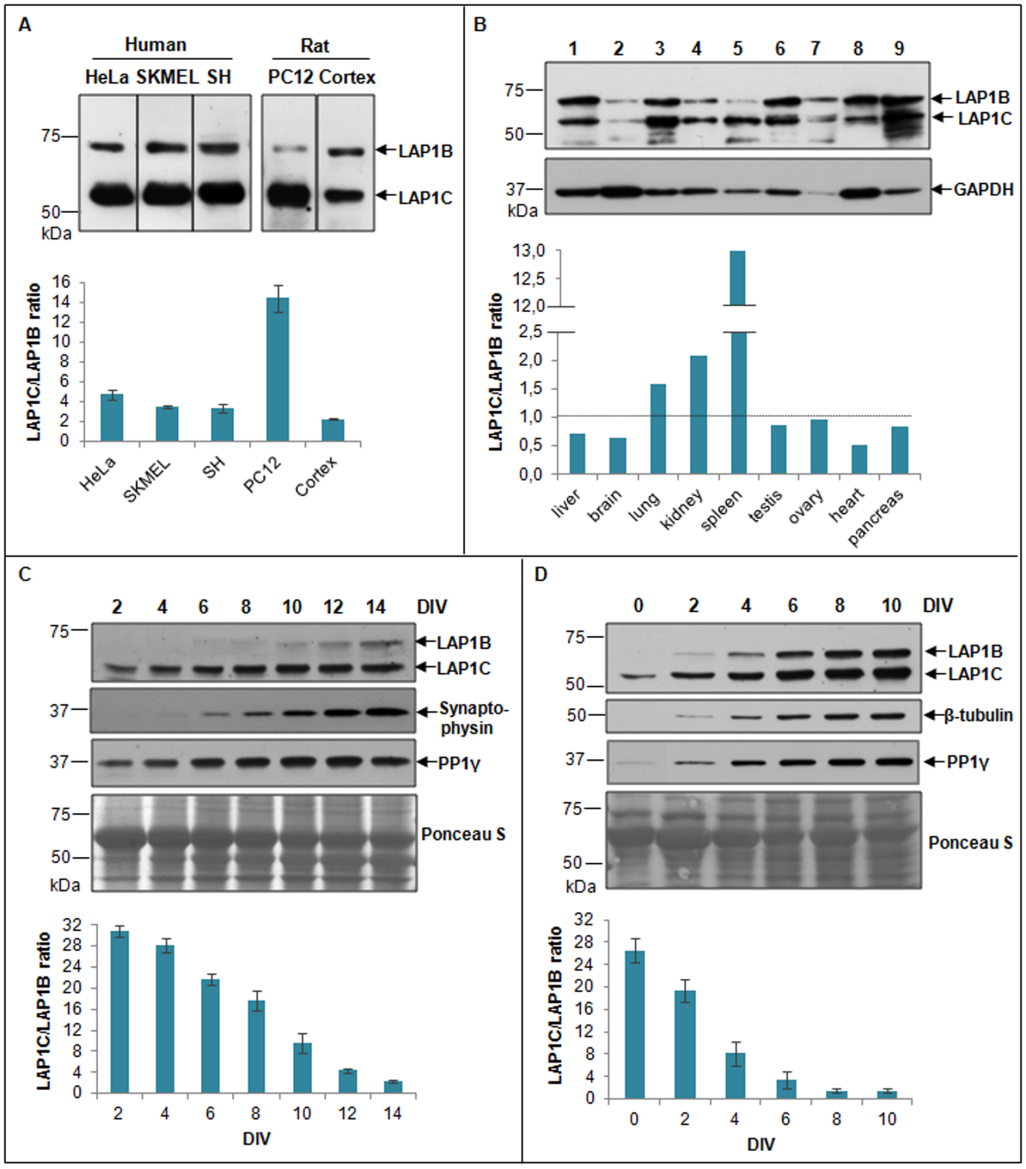
LAP1 expression in different cell lines and tissues. **A-** Endogenous LAP1 isoforms were detected in HeLa, SKMEL-28 and SH-SY5Y human cells and in rat PC12 cell line and rat cortex lysates. **B-** Human tissue blot (Clontech) was immunoblotted with LAP1 antibody. 1, liver; 2, brain; 3, lung; 4, kidney; 5, spleen; 6, testis; 7, ovary; 8, heart, 9, pancreas. **C-** Endogenous expression of LAP1 isoforms was detected in primary cortical neurons for 14 DIV, using a LAP1 antibody. Synaptophysin and PP1γ were used as controls for expressing patterns. Ponceau S staining was used to check equal loading. **D-** Endogenous expression of LAP1 isoforms was detected in SH-SY5Y cells differentiated with retinoic acid for 10 DIV using a LAP1 antibody. β-tubulin and PP1γ were used as controls. Ponceau S staining was used to check equal loading.

It was previously reported that rat LAP1A is the major isoform identified in rat liver tissue, while LAP1C is highly expressed in cultured cells [4]. Therefore, immunoblotting with LAP1 antibody in human samples was performed, in order to establish if human LAP1 isoforms are differentially expressed in human cell lines and distinct tissues. In fact for the different human cell lines (HeLa, SKMEL-28 and SH-SY5Y cells, derived from a cervical cancer, skin melanoma and neurosblatoma, respectively) tested, LAP1C protein is more abundant than LAP1B (Figure 8A), in agreement with previous reports. In rat, LAP1C is the major isoform in the pheochromocytoma rat cell line PC12, while in rat cortex lysates, the ratio between LAP1C and LAP1B decreases (Figure 8A), although in the latter case expression of both isoforms is quite similar. In contrast, LAP1B and LAP1C expression profiles, in human tissues, appear to be dependent on the specific tissue (Figure 8B). LAP1C has higher expression levels in lung, kidney and spleen, compared to LAP1B. In contrast, LAP1B is the major isoform present in liver, brain and heart, while in ovary, testis and pancreas the expression of both LAP1B and C is quite similar. An interesting aspect is the fact that in human brain, the expression of LAP1B is higher than LAP1C. Other bands appear in these blots and their significance deserves further attention.

Previous reports suggested that the expression of the three mouse LAP1 isoforms seems to be developmentally regulated. By comparing the mouse P19 teratocarcinoma cell line and the differentiated P19MES line, mouse LAP1A and LAP1B were strongly expressed only in the differentiated cells, while LAP1C was found in both cell types [5]. Therefore, we also analyzed the expression pattern of LAP1 isoforms during the establishment of cortical primary cultures for 14 days *in vitro* (DIV). Our data showed that LAP1B and LAP1C expression increases during neuronal development (Figure 8C). However, LAP1C expression in cortical neurons reaches a maximum a 10 DIV and remains almost constant thereafter. In contrast, LAP1B is expressed at very low levels until 10 DIV and increases over 14 DIV (Figure 8C). LAP1B is barely detected at 2 and 4 DIV, in comparative terms (longer exposure times will produce more intense bands). The pre-synaptic marker synaptophysin and PP1γ were used as controls. Ponceau S staining was used to confirm that equal amount of protein was loaded on each of the wells (Figure 8C). These results indicate that LAP1B is highly expressed in functional mature neurons since its intracellular levels correlate very well with synaptophysin levels, a pre-synaptic marker.

Similar results were obtained when SH-SY5Y cells were differentiated. Briefly, SH-SY5Y cells were plated at a density of 1×10^5^ and grown for 10 days in MEM/F12 medium with 10% FBS in the presence of 10 µM retinoic acid. Under the experimental conditions tested, the expression of both LAP1B and LAP1C increased during differentiation (Figure 8D). However the increases of LAP1B levels were more marked than LAP1C levels, as demonstrated by the ratio between both proteins (Figure 8D) and its intracellular levels are high when the cells are differentiated. Of note, undifferentiated SH-SY5Y cells also express the LAP1B isoform and it was visible when membranes were exposed for longer periods of time. Ponceau S staining was used to confirm equal protein loading on the gel (Figure 8D).

### Regulation of both isoforms by post-translational modifications

**Figure 9:**
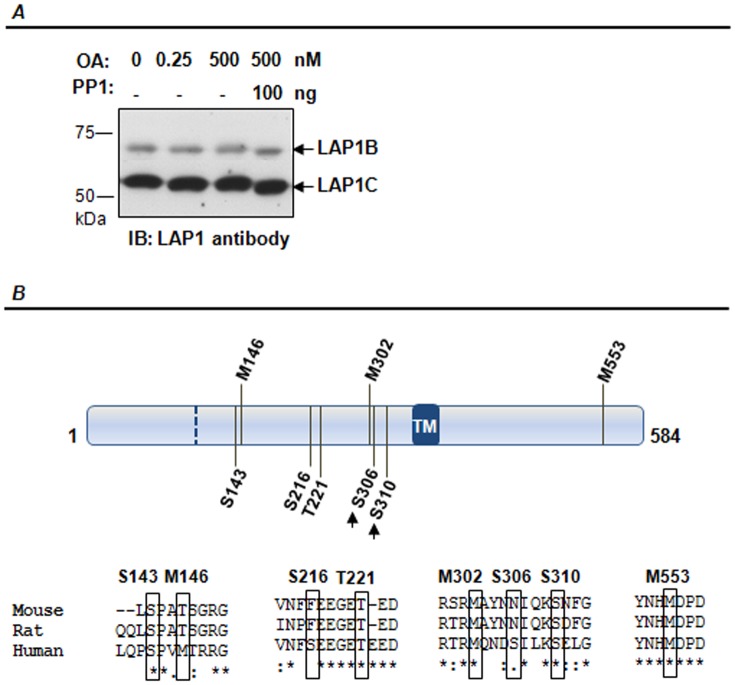
Human LAP1 post-translational modifications. **A-** SH-SY5Y cells were incubated with 0, 0.25 or 500 nM okadaic acid (OA) for 3 hours. Lysates were incubated at 30°C for 1 hour with or without 100 ng of PP1γ1 protein. **B-**. LAP1 isoforms are phosphorylated on Ser 143, Ser 216, Thr 221, Ser 306 and Ser 310 residues. PP1 was found to be responsible for Ser 306 and Ser 310 residues dephosphorylation (indicated by arrows). Methionine residues 146, 302 and 553 residues were found to be oxidized. Post-translational modified residues and flanking regions were aligned against others species using ClustalW algorithm. The residues numeration is relative to the LAP1B sequence and the translation initiation site of LAP1C is indicated by a dashed line. IB, immunoblotting.

We have recently reported that human LAP1B is dephosphorylated *in vitro* by PP1 [15]. Protein phosphorylation is a crucial mechanism for signal transduction that regulates the biological activity of diverse proteins [35], [36]. Thus, it is important to understand if human LAP1C is likewise regulated by protein phosphorylation and if PP1 is responsible for its dephosphorylation, as occurs with LAP1B. Hence we performed an assay similar to that previously reported by us and developed for LAP1B [15]. Hence, SH-SY5Y cells were incubated with two different concentrations of OA (0.25 nM and 500 nM that inhibit PP2A and PP1/PP2A, respectively) and cell lysates were further incubated with 100 ng of recombinant purified PP1γ1 protein (Figure 9A). The results showed that after addition of purified PP1γ1 an increase in the migration of both LAP1 isoforms is detected (Figure 9A), consistent with the dephosphorylation of these proteins by PP1γ1. Therefore, it appears that both human LAP1B and LAP1C are desphosphorylated by PP1.

Further, HPLC-MS analysis unequivocally showed that both isoforms are regulated by protein phosphorylation. SH-SY5Y cells were incubated with 0.25 nM OA or 500 nM OA. A control; cells not treated with OA, was also included in the experiment. These cells were lysed and immunoprecipitated with LAP1 specific antibody. Immunoprecipitates were loaded on SDS-PAGE and 68 kDa and 56 kDa bands (corresponding to LAP1B and LAP1C, respectively) were excised and subsequently analysed by nanoHPLC-MS in a Q Exactive mass spectrometer. In total, four phosphorylated residues (Ser216, Ser306, Ser310, and Thr221) were identified in the peptides resultant from digestion of LAP1C protein (Figure 9B). Since LAP1B protein sequence is equal to LAP1C, with the exception of a longer N-terminal, we infer that LAP1B could also be phosphorylated at the same residues. Thus, the numeration of the residues is relative to the human LAP1B protein sequence for comparative analysis. Peptides phosphorylated on Ser216 and Thr221 residues were identified in the control condition (without OA treatment) and also when PP1 was inhibited (500 nM OA treatment). Moreover, two additional residues, Ser 306 and Ser310, were found to be phosphorylated specifically in the latter condition (Figure 9B). These results demonstrated that PP1 is likely to be involved in the dephosphorylation of Ser306 and Ser310, since these residues were not found to be phosphorylated in the control condition (without OA treatment) nor in the PP2A-inhibited condition (0.25 nM OA treatment). Further, a membrane enriched fraction from SH-SY5Y cells, acquired as previously described, was also loaded on SDS-PAGE and the LAP1B and LAP1C bands analysed by HPLC-MS following the procedures described above. This analysis resulted in the identification of one additional phosphorylated LAP1B/C residue: Ser143 (Figure 9B). Overall, 5 different phosphorylated residues of LAP1B/C were identified, but only Ser306 and Ser310 residues were associated with PP1 activity. Furthermore, Ser143, Thr221 and Ser310 residues are conserved between human, rat and mouse species while Ser216 and Ser306 are not conserved (Figure 9B).

Additionally, another post-translational modification of human LAP1B and LAP1C was identified by HPLC-MS. Three methionines (Met146, Met302 and Met553) were modified by the addition of oxygen (Figure 9B). None of these methionines serves as an initiation site for LAP1B or LAP1C proteins synthesis. Furthermore, Met146 residue is not conserved, but Met302 and Met553 residues are conserved between human, rat and mouse species (Figure 9B).

## Discussion

The work here presented permitted the identification of a novel LAP1 isoform (LAP1C) in human cells using a variety of methods and the novel isoform was confirmed by HPLC-MS analysis. LAP1C has a putative short N-terminal domain, when compared to LAP1B. Furthermore, it appears that human LAP1 isoforms (LAP1B and LAP1C) are regulated at the transcriptional level and can be subject to post-translational modifications, namely protein phosphorylation and methionine oxidation. Additionally we established that PP1 mediates the dephosphorylation of LAP1C and B at Ser 306 and Ser 310 residues.

The genomic structure of *TOR1AIP1* genes (genes coding for LAP1) from human, rat and mouse species are conserved. Indeed, human and rat *TOR1AIP1* genes have 10 conserved exons, while mouse has 12 exons in total and 9 exons present high identity compared to rat and human exons (Figure 1). Additional exons 1b, 3b and 5b were found in mouse transcripts. Alignment of these exons against the human and rat genomic sequences revealed the presence of putative alternative exons 1b and 3b in the respective genes. However, we were not able to identify alternative exons in human transcripts by RT-PCR (Figure 3). The *in silico* data suggests that potential new isoforms of LAP1 could be generated by alternative splicing. In eukaryotes, transcript variants arise from a combination of alternative transcription start sites selection, alternative splicing and differential usage of polyadenylation sites [37]. It was previously reported that rat LAP1 isoforms are generated by alternative splicing [5]. In addition to alternative splicing, our data also suggests that alternative transcription start sites or alternative promoters could generate distinct LAP1 isoforms. Non-RefSeq mRNAs and ESTs in GenBank that lack the 5′ end region of exon 1, were found and these do not possess the first translation start site present in the full-length exon 1- transcripts. This is common to the three species analyzed (human, rat and mouse) and supports our thesis of the novel human LAP1C isoform. A significant number of genes use one or more alternative promoters. The usage of alternative promoters can induce alterations in the N-terminal of the protein coding sequence or create alternative ORFs, thereby potentiating the diversity of eukaryotic genome expression [38]–[40]. Moreover, alternative promoters are also functionally correlated with alternative splicing. Thus alternative promoter usage and alternative splicing are two major mechanisms for increasing transcript diversity [41], [42].

Although we were unable to produce an additional LAP1 human transcript by RT-PCR (Figure 3) or 5′RACE (data not shown) we showed that an alternative LAP1 transcript exists in human cells given that: (i) transfecting SH-SY5Y cells with two specific LAP1 shRNAs resulted in the knockdown of two LAP1 immunoreactive bands (68 and 56 kDa) (Fig. 2); (ii) the lower band of 56 kDa has the same molecular weight as the rat LAP1C isoform and HA-LAP1C co-migrates with the endogenous LAP1C at 56 KDa; (iii) Northern blot analysis revealed the presence of two differently sized RNAs (Fig. 4); (iv) alternative exons were found by *in silico* analysis. Furthermore and conclusively, the SDS-PAGE extracted 56 kDa protein (LAP1C), when analyzed by HPLC-MS, did not have peptides mapping N-terminal of exon 1 it contrasts dramatically with data found for LAP1B. A methionine at position 122 (relative to LAP1B sequence) was identified, indicating potential start codon for LAP1C translation (Table 1). The non-RefSeq mRNAs present in GenBank have differing N-terminal sequences thus supporting the existence of different LAP1 isoforms. Consistently, the downstream in frame putative start codon is present at position 122, consistent with the HPLC-MS results here presented. Thus, the theoretical molecular weight of the identified LAP1C isoform (Table 2) is similar to the 56 kDa band identified by immunoblotting using the LAP1 antibody. Moreover, HA-tagged LAP1C was expressed in human cells (SH-SY5Y) and co-migrated with the endogenous LAP1C as a band of approximately 56 kDa (Figure 6A). The immunolocalization of this novel isoform (Figure 6B) indicated that it is mainly localized in the nuclear envelope and also in the nucleus, in a manner similar to LAP1B [15]. However, additional co-localization studies of both isoforms should be performed to clearly determine if LAP1C and LAP1B present any localization differences.

Previous reports showed that deletion of a part of the nucleoplasmic domain of LAP1B increases the solubilization of this protein after detergent addition [6]. Hence, the resistance of the LAP1 isoforms (in particular LAP1C) to extraction from nuclear membranes was tested, using triton X-100 and increasing salt concentrations (NaCl). We demonstrated that LAP1C is more easily solubilized (Figure 7) than LAP1B, in agreement with the observation that human LAP1C differs from LAP1B in the 5′ end region of the first exon located in the nucleoplasmic domain. Although it becomes clear that human LAP1 isoforms putatively contain different N-termini, the origin of these proteins has to be established and the complete sequence of LAP1C determined using the N-terminal sequencing methodology.

Interestingly, a recent report by Kayman-Kurekci et al. [43] showed for the first time that a mutation in the *TOR1AIP1* gene at the N-terminal region completely abolishes the expression of LAP1B. This mutation is responsible for a form of muscular dystrophy. Of note, in the western blots performed, the protein band corresponding to the LAP1B protein was absent but another band higher then 50 KDa was evident, which the authors stated as a putative additional LAP1 isoform present in endomysial cells [43].We strongly believe that this isoform corresponds to LAP1C, which is here described in human cells for the first time.

The identification of two human LAP1 RNAs by Northern blot analysis and the existence of non-RefSeq mRNAs matching with the putative LAP1C sequence in GenBank, suggests that LAP1B and LAP1C are products of different RNAs and thereby their generation is regulated at the transcriptional level. However, the two RNAs detected appear have similar abundance in SH-SY5Y cells (Figure 4), which is not in direct proportion to the protein levels of LAP1B and putative LAP1C isoforms detected by immunoblotting. In fact, the abundance of an mRNA transcript may only partially predict the protein abundance. Moreover, the concentration of a protein not only depends on the mRNA concentration but also depends on the translational efficiency and degradation of the protein [44], [45]. Nevertheless, given the presence of two distinct RNAs, it is plausible that the LAP1 isoforms could arise from alternative splicing or alternative promoter usage and consequently use an alternative transcription initiation site. Database searches for alternative promoters, identified an upstream putative LAP1C translation initiation site (Figure 5). Despite this, resolution of this question will require additional experiments. Several reports showed that the 5′ UTR region is shorter in certain mRNAs and arise via alternative splicing or activation of a downstream alternative promoter. Normally, this process leads to the increased synthesis of a specific protein, meaning that the translation of short 5′ UTR mRNAs is more efficient in those cases. On the other hand, extension of the 5′ UTR may provide a more complex and controlled regulation of gene expression [41], [46], [47]. It will also be interesting to understand the consequences in the lost of the N-terminal domain of LAP1C in protein-protein interactions. Previous reports suggested that rat LAP1C has a weaker interaction with the nuclear lamina in comparison with rat LAP1A and LAP1B [4], [9]. Moreover, rat LAP1A and LAP1B were found to bind directly to lamins A, C and B1 *in vitro* and probably indirectly to chromosomes [9], while rat LAP1A/C was found to immunoprecipitate with B-type lamins [48].

We have recently reported that LAP1B is dephosphorylated *in vitro* by PP1 [15]. Protein phosphorylation is a major signaling mechanism in eukaryotic cells that is able to regulate the biological activity of diverse proteins [35], [36], including proteins involved in pathological conditions [18], [49]–[51]. In the work here described, the newly identified human LAP1C isoform was shown to be also dephosphorylated by PP1 (Figure 9). In addition, phosphorylation sites were mapped by HPLC-MS. Five phosphorylated residues were identified (Ser143, Ser216, Thr221, Ser306 and Ser310) in LAP1B/LAP1C and PP1 was shown to be associated with the dephosphorylation of two of these residues (Ser306 and Ser310, the numeration of the residues is relative to the human LAP1B protein sequence). Moreover, Ser143, Thr221 and Ser310 residues are conserved between the species analysed. Functional phosphorylation sites are more conserved during evolution than sites without characterized function [52]. Therefore, it will be interesting to understand the effects of reversible protein phosphorylation on LAP1 function. In addition, we found that LAP1 is post-translationally modified by methionine oxidation. Methionine is oxidized to methionine sulfoxide by the addition of an extra oxygen atom (methionine oxidation) and this process may function as a protein regulatory mechanism [53].

LAP1 is ubiquitously expressed in neuronal and non-neuronal tissues [11], [54]. Previous reports showed that LAP1A, B and C are all detected in liver, spleen, brain and kidney rat tissues, but LAP1A seems to be the major isoform present in rat liver. In contrast, LAP1C is the major isoform found in rat cultured cells [4]. Our results are in agreement with previous data, since we detected higher expression of human LAP1C isoform in different cultured cells in comparison to LAP1B (Figure 8A). However, when human tissues were analyzed, it was found that the expression of LAP1B/LAP1C is dependent on the specific tissue (Figure 8B). Nevertheless, the human LAP1 isoform with higher molecular weight identified so far, LAP1B, is more abundant in liver than the smallest LAP1C isoform, in agreement with previous studies in rat liver tissue. Of particular interest is the profile of LAP1 expression in brain. LAP1 was found to interact with torsinA, the protein involved in the neurological disorder DYT1 dystonia. LAP1 is able to relocate torsinA to the NE, although the latter is normally found in the ER [11]. The torsinA mutant form, which is present in DYT1 dystonia patients, is also primarily relocated to the NE [55], [56]. Therefore, it was suggested that abnormal accumulation of torsinA in the nuclear envelope may lead to a nuclear envelope dysfunction through LAP1 binding, thus contributing to DYT1 dystonia pathogenesis.

Furthermore, the expression of LAP1 isoforms seems to be regulated during mouse spinal cord development and maturation [57]. In order to understand if LAP1 isoforms are also differentially regulated in neuronal models, its expression profile during neuronal differentiation of SH-SY5Y cells (0–10 DIVs) (Figure 8D) and during maturation of rat cortical primary cultures (2–14 DIVs) (Figure 8C) was analyzed. Thus, we showed that in both models, LAP1C is already detected at 0/2 DIV while LAP1B is barely detected at this time point. However, the expression differences between LAP1C/LAP1B decrease during DIVs, which means that LAP1B is upregulated later when compared to LAP1C. Moreover, the expression of LAP1C is maintained at late DIVs while LAP1B continues to increase. Previous studies reported that LAP1A and LAP1B are strongly expressed in mouse liver cells and differentiated P19MES cells, while in mouse embryonic 3T3 cells and undifferentiated P19 cells LAP1A and LAP1B are barely detected. In contrast LAP1C is expressed in all cells [5]. In our work, we provide a link between LAP1 expression and the state of neuronal differentiation of SH-SY5Y cells and maturation of neurons, suggesting that LAP1 may play a role in those processes. It was previously reported that knockout of *TOR1AIP1* gene in mouse results in perinatal lethality and the nuclear membranes of various tissues and cultured neurons of those mouse embryos showed severe abnormalities [54]. Therefore, disturbances in the expression of LAP1 may compromise neuronal survival.

In conclusion, this is the first report of human LAP1C isoform recovery from human cells. Some related mRNA sequences have been already described in GenBank, however they were not identified as splice variants of human LAP1. Moreover, this work provides new insights with respect to *TOR1AIP1* genomic structure, potential transcripts and protein isoforms. Our data suggest that new potential human LAP1 isoforms could be generated by alternative splicing and or alternative start sites and deserves further investigation. In closing, it is evident that human LAP1B and LAP1C isoforms are differentially expressed and post-translationally regulated by protein phosphorylation and methionine oxidation. Finally, it was shown that PP1 is likely involved in the dephosphorylation of at least two LAP1B/LAP1C residues (Ser306 and Ser310).

### Supplementary methods

#### *In vitro* translation (IVT)

LAP1B was generated by *in vitro* translation (IVT) from pET-LAP1B expression vector using the TnT-coupled transcription/translation kit (Promega), according to the manufacturer's instructions.

## Supporting Information

Figure S1 **Schematic representation of LAP1 non-RefSeq transcripts.** The translation initiation codons (ATG) and the stop codons (TAA or TGA in human and mouse/rat sequences, respectively) are indicated in each transcript.
(TIF)

Figure S2 ***In vitro*** **translation (IVT) of LAP1B.** IVT of pET-LAP1B generates only His-tagged LAP1B.
(TIF)

Figure S3 **Coomassie blue colloidal staining.** Total cell lysates, membrane containing-fraction and LAP1 immunoprecipitates (IP) were loaded on SDS-PAGE and the gel was further stained with Coomassie blue colloidal. After staining, the bands including the LAP1B (68 kDa) and LAP1C (56 kDa) proteins were excised and further analyzed by HPLC-MS.
(TIF)

Table S1 **Intron/exon junctions in the** ***TOR1AIP1*** **gene.**
(DOCX)

Table S2 **LAP1 peptides identified by mass spectrometry analysis.**
(DOCX)
