# Supplementary figures and images for: Identification of a Novel Human LAP1 Isoform That Is Regulated by Protein Phosphorylation
Source: PLoS One. 2014 Dec 2;9(12):e113732. doi: 10.1371/journal.pone.0113732 (PMC4252041; doi:10.1371/journal.pone.0113732)

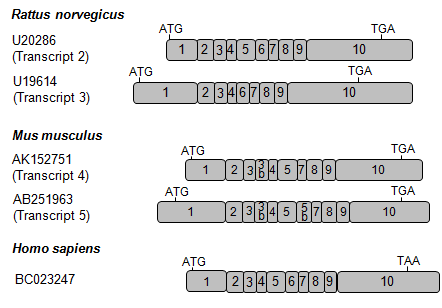

Supplement: Figure S1 — Schematic representation of LAP1 non-RefSeq transcripts. The translation initiation codons (ATG) and the stop codons (TAA or TGA in human and mouse/rat sequences, respectively) are indicated in each transcript. (TIF) [file pone.0113732.s001.tif]

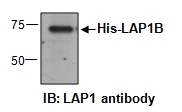

Supplement: Figure S2 — In vitro translation (IVT) of LAP1B. IVT of pET-LAP1B generates only His-tagged LAP1B. (TIF) [file pone.0113732.s002.tif]

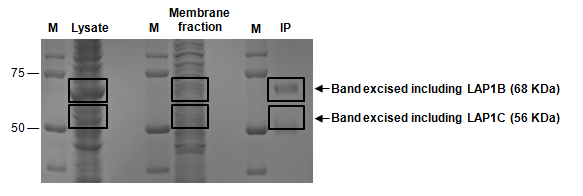

Supplement: Figure S3 — Coomassie blue colloidal staining. Total cell lysates, membrane containing-fraction and LAP1 immunoprecipitates (IP) were loaded on SDS-PAGE and the gel was further stained with Coomassie blue colloidal. After staining, the bands including the LAP1B (68 kDa) and LAP1C (56 kDa) proteins were excised and further analyzed by HPLC-MS. (TIF) [file pone.0113732.s003.tif]
