# Supplementary material for: Identification of a Novel Human LAP1 Isoform That Is Regulated by Protein Phosphorylation
Source: PLoS One. 2014 Dec 2;9(12):e113732. doi: 10.1371/journal.pone.0113732 (PMC4252041; doi:10.1371/journal.pone.0113732)
Supplement: Table S1 — Intron/exon junctions in the TOR1AIP1 gene. (DOCX) [file pone.0113732.s004.docx]

**Table S1. Intron/exon junctions in the *TOR1AIP1* gene**

|  | **Intron 3’end** | **5’end – Exon size (bp) – 3’end** | **Intron 5’end** |
| --- | --- | --- | --- |
| **Exon 1** |  |  |  |
| Human |  | ACTGCCTC – 936 - CTCTGAAG | GTgaggac |
| Mouse |  | CGG*AG** - 723 - *CGCG*** | **a*cagt |
| Rat |  | *AAA*ACA – 672 – *CGC*G** | **a*cagt |
| **Exon 1b** |  |  |  |
| *Human |  | ATAGTTTG - 92 - TCGTGAAG | GTactgac |
| Mouse |  | GA*CAGGA – 137 – *TT**G*T | ***ag**g |
| *Rat |  | AA*GAGGA – 136 - *TT**A*T | ***ac**g |
| **Exon 2** |  |  |  |
| Human | ttttttAG | AGGATGAA – 78 - GGCTCCAG | GTaagaat |
| Mouse | *gc*g*** | ******** – 75 - ****T*** | *******g |
| Rat | *gc*a*** | ******** - 75 - *A**T*** | *******t |
| **Exon 2b** |  |  |  |
| *Human | ttctctAG | CTGCTGTA – 131 - AAAGAAGG | GTacttga |
| Mouse | - | - | - |
| Rat | - | - | - |
| **Exon 3** |  |  |  |
| Human | tatattAG | CAGTGAGT – 60 - AAGATATG | GTaagaga |
| Mouse | **c*cc** | G*TGA*TG – 60 - ****CT** | ******t* |
| Rat | **t*cc** | TGATG*GT – 60 - ****CC** | ******g* |
| **Exon 3b** |  |  |  |
| *Human | tcttgcAG | ATTCGACA – 45 - TTAATTAT | GTtcgttt |
| Mouse | ****a*** | ****A*** – 45 – *G**AG*G | ***t**** |
| *Rat | ct*ata** | **C*A*** - 45 – GC*CAAGT | **GT**GC |
| **Exon 4** |  |  |  |
| Human | tcttctAG | AAGCCACC – 42 - TGAAGAAG | GTatttta |
| Mouse | ******** | ****T*CC – 39 – C******* | ***a***c |
| Rat | ******** | *C**TT** - 39 – GC*GTTT* | ***a***t |
| **Exon 5** |  |  |  |
| Human | ctctttAG | GAGAAAAC – 87 - ATCTGGAG | GTaatatt |
| Mouse | *ct*g*** | ******C* - 81 – G**AA*** | *****g** |
| Rat | *ct*g*** | ******C* – 81 – G***A*** | ****ca** |
| **Exon 5b** |  |  |  |
| Human | - | - | - |
| Mouse | tggtttAG | ATGAAGCC - 56 - GCCACACA | GTtaagta |
| Rat | - | - | - |
| **Exon 6** |  |  |  |
| Human | atttttAG | ATAAAACC – 57 - ATCACAAA | GTaagtaa |
| Rat | g****c** | **G**G** – 57 - *C****C* | *******c |
| Mouse | - | - | - |
| **Exon 7** |  |  |  |
| Human | tgtttcAG | GTCAAAAC – 42 - AGTGCTAA | GTaagtag |
| Mouse | ****ct** | A***GG** – 42 - *C****G* | ******tc |
| Rat | ****ct** | ***GGGGG – 45 - *C****GC | ******gc |
| **Exon 8** |  |  |  |
| Human | tgcttcAG | GCTCAGGA – 69 - GATGCAAA | GTaagtag |
| Mouse | ******** | C******* - 69 – A***GCGT | ******** |
| Rat | ******** | C*A***** - 69 – A***GC*T | ******** |
| **Exon 9** |  |  |  |
| Human | accattAG | ATGGCAGC – 57 - CAGCCGAC | GTaagttt |
| Mouse | tatt**** | **AA**A* – 57 - ***G*C** | ******c* |
| Rat | tatt**** | **AA**A* – 57 - ***G*A*G | ******** |
| **Exon 10** |  |  |  |
| Human | ctgagtAG | AAGTGACT – 2624 - ATACTCTC |  |
| Mouse | *****c** | **TC*G** – 3111 - **GT*A*A |  |
| Rat | *****c** | **GCAG** – 1547 - *AAG*T*T |  |

Nucleotides sequences of the 5’ and 3’ end of exons and the donor and acceptor sites at the intron/exon junctions (in upper case) of the human, mouse and rat *TOR1AIP1* genes are deduced from the *TOR1AIP1* genomic sequences. Sequences of intron ends are given in lower case, except for the donor and acceptor sites in upper case. Exons and introns are numbered as referred in Fig. 1 and exon sizes are given. Homologies between human and mouse/rat sequences are shown by asterisks. Exon 1b and 3b in human and rat sequences and exon 2b in the human sequence are putative exons.
