# Supplementary material for: Identification of a Novel Human LAP1 Isoform That Is Regulated by Protein Phosphorylation
Source: PLoS One. 2014 Dec 2;9(12):e113732. doi: 10.1371/journal.pone.0113732 (PMC4252041; doi:10.1371/journal.pone.0113732)
Supplement: Table S2 — LAP1 peptides identified by mass spectrometry analysis. (DOCX) [file pone.0113732.s005.docx]

**Table S2. LAP1 peptides identified by mass spectrometry analysis**

|  | **68 kDa band (LAP1B)** | | | **56 kDa band (LAP1C)** | | |
| --- | --- | --- | --- | --- | --- | --- |
| **Peptides** | **Lysate** | **Membrane fraction** | **IP** | **Lysate** | **Membrane fraction** | **IP** |
| EGWGVYVTPR |  | x | x |  |  |  |
| LAPQNGGSSDAPAYR |  |  | x |  |  |  |
| FSDEPPEVYGDFEPLVAK |  | x | x |  |  |  |
| LQQQHSEQPPLQPSPVMTR | x | x |  | x | x | x |
| LQQQHSEQPPLQPSPVMTRR |  |  |  |  | x |  |
| DSHSSEEDEASSQTDLSQTISK |  | x | x |  |  | x |
| DSHSSEEDEASSQTDLSQTISKK |  |  | x |  |  | x |
| SIQEAPVSEDLVIR | x | x | x | x | x | x |
| RPPLRYPR |  |  |  | x | x |  |
| VNFSEEGETEEDDQDSSHSSVTTVK |  | x | x | x |  | x |
| SSSQYIESFWQSSQSQNFTAHDK |  |  |  |  |  | x |
| QPSVLSSGYQK |  |  | x |  |  | x |
| TPQEWAPQTAR |  |  |  |  | x | x |
| TRMQNDSILKSELGNQSPSTSSR |  |  |  |  | x |  |
| MQNDSILKSELGNQSPSTSSR |  |  |  | x | x | x |
| QVTGQPQNASFVK | x | x | x | x | x | x |
| QVTGQPQNASFVKR |  | x | x | x | x | x |
| NKYQGQDEKLWK |  |  |  | x | x |  |
| SQPAILLLTAAR |  | x | x |  | x | x |
| SQPAILLLTAARDAEEALR |  |  |  | x | x |  |
| IDGTDKATQDSDTVKLEVDQELSNGFK |  |  |  |  |  | x |
| ATQDSDTVKLEVDQELSNGFK |  | x | x |  | x | x |
| LEVDQELSNGFK |  |  | x |  | x | x |
| FESFPAGSTLIFYK |  | x | x |  | x | x |
| DVALVLTVLLEEETLGTSLGLK |  |  |  |  |  | x |
| FTNSNTPNSYNHMDPDKLNGLWSR |  |  |  |  | x |  |
| ISHLVLPVQPENALKR | x | x |  | x | x | x |
| ISHLVLPVQPENALK | x | x |  | x | x | x |
| **Nº peptides** | 5 | 13 | 14 | 11 | 18 | 19 |

Three different samples were analyzed by mass spectrometry analysis: total cell lysates, membrane containing-fraction and LAP1 immunoprecipitates (IP), as described in the methods section. Samples were loaded on SDS-PAGE, gels stained with Coomassie blue colloidal and 68 kDa and 56 kDa bands (corresponding to LAP1B and LAP1C, respectively) excised from the gel before mass spectrometry analysis. The different peptides identified are listed on the right and the presence of the peptides in each condition analyzed (lysate, membrane fraction or IP) is marked by an x. The number of peptides identified in each condition is also shown. In total, 80 unique peptides were identified, taking in account all conditions analyzed.
